# Supplementary material for: Multi-strain probiotics (Hexbio) containing MCP BCMC strains improved constipation and gut motility in Parkinson’s disease: A randomised controlled trial
Source: PLoS One. 2020 Dec 31;15(12):e0244680. doi: 10.1371/journal.pone.0244680 (PMC7774928; doi:10.1371/journal.pone.0244680)
Supplement: S1 File — (DOCX) [file pone.0244680.s002.docx]

UNIVERSITIKEBANGSAANMALAYSIA

Hospital Canselor Tuanku Muhriz, PPUKM

**THE EFFECTIVENESS OF PROBIOTICS ON CONSTIPATION IN PARKINSON DISEASE: RCT**

DR. AZLIZA BINTI IBRAHIM

PRINCIPLE SUPERVISOR:

PROFESSOR DATIN DR. NORLINAH MOHAMED IBRAHIM

CO-SUPERVISORS

ASSOCIATE PROFESSOR DR. RAJA AFFENDI RAJA ALI

STATISTICAL ANALYSIS SUPERVISOR

PROFESSOR DR MOHD RIZAL ABDUL MANAF

**TABLE OF CONTENTS**

**1. INTRODUCTION** 1

**2. LITERATURE REVIEW** 4

**3. THESIS STATEMENT** 6

3.1 RESEARCH QUESTION 6

3.2 RESEARCH HYPOTHESIS 6

**4. OBJECTIVE** 6

4.1 PRIMARY OBJECTIVE 6

4.2 SECONDARY/SPECIFIC OBJECTIVE 6

**5. RESEARCH METHODOLOGY** 7

5.1 STUDY DESIGN 7

**6. HOSPITAL PROFILE/DEMOGRAPHIC** 7

**7. STUDY PERIOD** 8

**8. SUBJECTS** 8

8.1 TARGET POPULATION 8

**9. SAMPLING** 9

9.1 INCLUSION CRITERIA 9

9.2 EXCLUSION CRITERIA 10

**10. DATA COLLECTION** 11

10.1 DATA COLLECTION TOOLS 11

10.2 STUDY PROTOCOL 12

10.3 STUDY ASSESSMENT 13

**11. STATISTICAL ANALYSIS** 16

11.1 SAMPLE SIZE CALCULATION 16

**12. STUDY FLOW CHART** 18

**13. ETHICAL ISSUES** 19

**14. WORKING SCHEDULE** 19

14.1 Gantt chart 19

**15. ESTIMATION OF COST** 20

16. **REFERENCES** 21

17. **APPENDICES** 25

**LIST OF ABBREVIATIONS**

A-Sync Alpha-synuclein

CFU Colony Forming Unit

GTT Gatrointestinal Transit Time

HLS Hard and Lumpy stool

MDS Movement Disorder Society

NMS Non-motor Symptoms

PD Parkinson Disease

PDQ39 39-Parkinson’s Disease Questionnaire

SCFA Short Chain Fatty Acids

SPSS Statistical Package for the Social Sciences Software

UPDRS Unified Parkinson’s disease Rating Scale

GQ Garrigues Constipation Questionnaire

**INTRODUCTION**

Constipation is one of the commonest non motor symptoms (NMS) in Parkinson Disease (PD) and reported in approximately 80-90% of patients ^1-3^. The NMS occur throughout the stages of PD, and some even predate the development of motor symptoms by 5-10years^4^. Constipation and digestive issues in PD are due to multifactorial factors contributed by the enteric and central nervous system dysfunction, side effects of medications and natural process of ageing. Besides, dystonia of the striated external sphincter and the reduction of dopamine containing neurons in the enteric nervous system also contributes towards the development of gastrointestinal symptoms in PD ^5^. A study by Azmin et al^6^ shows that NMS prevalence was as high as 97.3% in patients with idiopathic PD of which gastrointestinal symptoms were the commonest affecting 76.1% of patients. In general, constipation is diagnosed by the reporting of multiple symptoms such as infrequent BMs, production of hard or lumpy stools (HLS), incomplete evacuation, straining, abdominal discomfort or pain, and bloating. The management of constipation remains challenging. Bulking agents, osmotic laxatives, stimulant laxatives, and stool softeners are commonly used with most of the patients adopt self-management approaches with 80% trying over the counter product ^7^ such as foods believed to exert a laxative effect, “functional foods,” and nutraceuticals ^8^.

Recent studies showed that alpha-synuclein (a-Sync) aggregation in colonic mucosal of PD patients leads to disruption of intestinal barrier integrity and causes constipation^9^. Alpha-synuclein is a primary structural component of Lewy bodies (protein clumps) and has been shown to play role in sporadic/familial PD. More recently, studies have shown that there is a gut dysbiosis with alterations in fecal microbial composition among PD patients which also contributes toward constipation in PD. This gut dysbiosisis is believed to cause neuroinflammation leading to a-Sync misfolding in the colonic mucosal of PD patients.

A recent study by Keshavarzian et al^10^ compared colonic bacterial composition among PD patients and healthy controls utilising sigmoid mucosal biopsies and fecal samples. Fecal and mucosal associated microbiota composition were characterized using highthroughput ribosomal RNA gene amplicon sequencing and it was found that there were significant differences at all taxonomic levels in fecal and mucosal associated microbiota communities between PD patients and control. Anti-inflammatory butyrate producing bacteria from the genera *Blautia, Coprococcus,* and *Roseburia* were signiﬁcantly less abundant in PD fecal samples compared to control which could potentially have caused intestinal inflammation and barrier dysfunction ^11-12^ and possibly the aggregation alpha-Sync protein in colonic mucosal.

Another study on SCFA concentrations and microbiota composition by M.M. Unger et al^13^ in fecal samples of 34 PD patients and 34 age-matched controls, showed that fecal samples from PD patients had low concentration of short chain Fatty Acid (SCFA). The study also showed that SCFA bacterial *phylum Bacteroidetes* and the bacterial family *Prevotellaceae* were reduced but *Enterobacteriaceae* (Gram Negative Bacilli) were more abundant in fecal samples from PD patients compared to matched controls. Therefore it was suggested that the reduction in SCFA induced alteration in enteric nervous system contributing to gastrointestinal dysmotility^14^.

Forsyth et al^14^ reported an increased intestinal permeability in PD that correlated with staining for E. coli (a bacterial species assigned to the family *Enterobacteriaceae*) and concluded that a compromised intestinal barrier might help to expose the enteric nervous system to noxa in PD . Hence, the observed increased abundance of *Enterobacteriaceae* might be pathophysiologically relevant for gastrointestinal dysmotility in PD.

There are several types of SCFAs produced by metabolic activities of intestinal bacteria, including acetic, propionic, butyric, and lactic acids ^15^. Of these SCFAs, butyrate and lactate are of particular importance in mechanisms of constipation. Butyrate has been shown to have anti-inflammatory properties and is involved in many cellular processes such as repair of the gut mucosal lining, stimulation of the autonomic nervous system and production of hormones associated within the GI tract ^16^. Besides butyrate, lactic acid is another important SCFA produced by intestinal bacteria such as *lactobacilli, bifidobacteria, enterococci, streptococci and Eubacterium*^17^. Lactate in colonic mucosal is normally used by other butyrate-producing bacteria as a precursor to synthesis butyrate products^18^.Based on the role of SCFA-butyrate organism in colonic mucosal and the evidence of low abundance of butyrate producing organism in PD patients, there is a potential role for probiotics in correcting the imbalance of intestinal bacterial (dysbisosis) and enhancing the number of this good microbiota in improving digestive symptoms.

**LITERATURE REVIEW**

Probiotics are live microorganism that are able to promote healthy digestive tract and immune system and the benefits are widely studied at this time. When probiotics are administered in adequate amounts, it gives health beneﬁt to the host^19^. There are several potential mechanisms of action by which probiotics may beneﬁt functional constipation^20^. First, probiotics modify the gastrointestinal microbiota, which is known to be altered in constipation^21 -22^. Second, probiotic metabolites may alter gut function, including sensation ^23-24^ and motility ^25-26^. Third, some probiotics increase the production of lactate and short-chain fatty acids, reducing luminal pH, which some researchers have proposed will enhance colonic peristalsis and shorten whole gut transit time (GTT) ^27-28^.

A randomised double blind, placebo-controlled trial study on efficacy of probiotics and prebiotics in Parkinson disease with constipation by Michela et^29^ al showed that PD with constipation who consumed fermented milk containing multiple probiotic strains and prebiotic fibre increase the number of complete bowel movements significantly. In this study, patients were randomly assigned to either fermented milk, containing multiple probiotic strains (*Streptococcus salivarius subsp thermophilus, Enterococcus faecium, Lactobacillus rhamnosus GG, Lactobacillus acidophilus, Lactobacillus plantarum, Lactobacillus paracasei, Lactobacillus delbrueckii subsp bulgaricus, andBifidobacterium )* of 250x 10^9^ colony forming unit (CFU) and prebiotic fibre, or placebo, once daily for 4 weeks. The above findings support the theory that manipulation of gut microbiota maybe a therapeutic option for functional bowel disorders^17^.The study also showed that probiotic mixture with prebiotics had greater efficacy than single strains in assisting complete bowel movement in Parkinson disease, although it is not clear whether this was attributable to synergism or the consequence of the higher probiotic dose used in the study^30^.

A systematic review and meta-analysis of randomized controlled trials in unselected populations of patients with functional constipation demonstrated that the use of probiotics has substantial benefits in stool frequency, transit time and stool consistency with favourable beneficial effects of *B. lactis* strain in particular^31^. The results of this study indicate that, overall, probiotics positively affected all of these measures. Besides, several other cardinal symptoms of constipation also significantly improved for example symptoms such as bloating, sensation of incomplete evacuation, occurrence of hard stools, and ease of stool expulsion.

An open-label, randomized, controlled study by T.Sakai et al^32^ using daily intake of fermented milk for 3 weeks demonstrated that daily consumption of fermented milk containing *Lactobacillus casei strain Shirota (LcS)* significantly reduced the incidence of hard or lumpy stool by 50% in healthy subjects with moderate to severe constipation compared to placebo.

Considering the high prevalence of constipation among PD patients and the positive effects on probiotics alleviating constipation, we attempt to explore the effects of probiotics in PD patients with constipation compared to placebo. We hypothesize that probiotics may be able to partially correct the gut dysbiosis that occurs in PD and improve quality of life among PD patients.

**3. THESIS STATEMENT**

**3.1 RESEARCH QUESTION**

- Are probiotics effective in improving constipation in PD patients?

**3.2 RESEARCH HYPOTHESIS**

- Probiotics consumption improves constipation , shorten Gut Transit Time(GTT) and improve constipation-related symptoms and Quality of life

**4. OBJECTIVE**

**4.1 PRIMARY OBJECTIVE**

- To determine the effects of probiotics (multistrains of Lactobacillus and Bifidobacterium ) on constipation among Parkinson disease patients compared to placebo
- To determine the effects of probiotics on Gastrointestinal Transit Time (GTT) compared to placebo

**4.2 SECONDARY OBJECTIVE**

- To determine the effects of probiotics on overall quality of life compared to placebo
- To determine the effects of probiotics on disease severity and non-motor symptoms score.

**5. RESEARCH METHODOLOGY**

**5.1 STUDY DESIGN AND METHODOLOGY**

Randomized Double Blind, Placebo Controlled Study

Study type: Interventional

This is a randomized, double-blind, placebo-controlled clinical trial. Recruitment of Parkinson disease patients with constipation will be done from the neurology clinic HUKM. Patients are considered eligible if they meet the Rome III criteria for functional constipation^33^ (2 or more criteria as listed in the box below in at least 25% occasion for the last 3 months) and will be provided written informed consent. Standard therapy for constipation is Syrup lactulose (osmotic agents) which promote intestinal motility and secretion. The recommended dose of lactulose for adults is 15 to 30 mL once or twice daily. The use of syrup lactulose is not forbidden and patient will be advised to take syrup lactulose as per needed. Fecal occult blood testing will be used to screen for colorectal malignancy in all eligible patients prior to intervention as positive test patients will not be included in the study. After written informed consent given, patients’ demographic data were collected.

|  | **6. HOSPITAL PROFILE/DEMOGRAPHIC**  **7. STUDY PERIOD**  June 2018 – June 2019  **8. SUBJECTS**  **8.1 TARGET POPULATION**  Patients with Parkinson disease attending the neurology outpatients clinic who fulfil inclusion and exclusion criteria  **9. SAMPLING**  **9.1 INCLUSION CRITERIA**   1. Idiopathic PD diagnosed using UK PD Society Brain Bank Criteria 2. Diagnosis of constipation according to Rome III Functional Constipation Criteria 3. Written informed consent   **ROME III Diagnostic Criteria : Functional Constipation**   \| Functional Constipation \| \| --- \| \| Must include 2 or more of the following:  Straining *  Lumpy or hard stools*  Sensation of incomplete evacuation*  Sensation of anorectal obstruction/blockage*  Manual manoeuvres to facilitate defecation(digital evacuation, support of pelvic floor)*  <3 defecation per week*  ***≥25% of defecations**  Loose stool rarely present without use of laxatives  Insufficient criteria for IBS \|   Based on Longstreth GF et al.Gastroenterology 2006 ;130:1480-1491  **9.2 EXCLUSION CRITERIA**   1. Secondary Parkinsonism 2. Previous history of small and large bowel disease 3. Previous extensive gastrointestinal tract surgery 4. Positive stool occult blood in the last 1 month 5. Use of probiotics or antibiotics in 2 weeks before baseline visit 6. Underlying Dementia 7. Hypothyroidism 8. Diagnosed with major depression and on antidepressant 9. Lactose intolerance   10. Type 1 and Type 2 DM  **10. DATA COLLECTION**  **10.1 METHOD OF DATA COLLECTION**  Patients with idiopathic PD attending the neurology outpatients’ clinic will be invited to participate in the study. All subjects will be screen for constipation by using Rome III Criteria – Constipation Module. Written informed consent will be obtained from all subjects after screening inclusion and exclusion criteria. Baseline demographic data will be recorded into a structured questionnaire.  Screening assessment :  1) Constipation screening using Rome III Criteria – Constipation Module  2) Fecal occult blood testing - negative test will be included in the study  The eligible subjects will be assess on the first visit as below :  1) Constipation symptoms by using Garrigues Constipation module (GQ)  2) Quality of life by using PDQ39 Questionnaire (self-administered malay version )  3) Parkinson Disease severity by using MDS-UPDRS  4)Non motor symptoms of PD by using NMS scale  (All the above questionnaires are validated)  Subsequently, subjects will be instructed to consume 4 red carmine capsule(food colourant) and once the stool turn red in colour, Gatrointestinal Transit Time(GTT) will be measured and recorded. Subjects will then receive a 3g sachet of either probiotics (containing multistrains of lactobacillus and Bifidobacterium 30 Colony Forming Unit) or placebo twice-daily for a duration of 8 weeks. Once consumption completed, subjects need to consume again another 4 red carmine capsule and GTT will be measured and recorded again. Final assessment will be done once subjects completed all the probiotics or placebo given(week 8) and subjects need to be assess again on constipation symptoms, non-motor symptoms, quality of life and disease severity same as done at baseline using the same questionnaire.  **10.2 STUDY PROTOCOL**  Randomization  All patients who fulfill inclusion and exclusion criteria will be stratified based on age, sex and background social economics. Patients who are enrolled will be randomized into two treatment groups which a probiotics group who will receive probiotics (containing multistrains of lactobacillus and Bifidobacterium 30 Colony Forming Unit) or placebo using blocks randomization method, in blocks of 4. Patients and investigator will remain blinded to the treatment protocol.  Following randomization, patients will receive their assigned treatment from the pharmacy department, Hospital Canselor Tuanku Muhriz, PPUKM  Treatment  Active treatment group will receive a 3 gram sachet of probiotics containing multistrains of lactobacillus and Bifidobacterium 30 Colony Forming Unit x 10^9^ to be taken twice daily for 8 weeks.  Composition of probiotics:   \| Lactobacillus acidophilus \| 107mg \| \| --- \| --- \| \| Lactobacillus casei \| 107mg \| \| Lactobacillus lactis \| 107mg \| \| Bifidobacterium bifidum \| 107mg \| \| Bifidobacterium infantis \| 107mg \| \| Bifidobacterium longum \| 107mg \|   Placebo group will receive a 3g sachet of substance (powder form) that has no therapeutic effect to be taken twice daily for 8 weeks.  All test products will be concealed in sealed envelopes, to ensure blindedness  **10.3 STUDY ASSESSMENT**  All patients will be assessed at baseline and week 8 for improvement in constipation symptoms using the Garrigues Constipation Questionnaire^35^, Quality of Life by using self- administered PDQ39 questionnaire (malay version)^36^, disease severity by using MDS-UPDRS and non-motor symptoms by using NMS scale . The Gastrointestinal transit time will be assessed at baseline and week 8 using red carmine capsule. Compliance to treatment, side effects and clinical assessments will be performed by a blinded assessor at baseline and week 8 for both groups.  **Gastrointestinal Transit Time Protocol**  Following randomization, subjects will be instructed to ingest 4 red carmine capsule after last bowel opening. Red carmine capsule is a food colorant and non-toxic to body which is used to measure GTT. The time of ingestion will be recorded. GTT is measured from the time of red carmine capsules consumption and first appearance of red colored stool. The duration from time of ingestion and appearance of red colored stool (Gastrointestinal transit time) will be recorded by patient at baseline, and week 8. The use of laxatives is not forbidden, but patients are required to use as per needed. GTT was measured during pre and end treatment in both groups.  **Constipation-Related Symptom Assessment**  Constipation related symptoms will be assessed using the Garrigues Constipation Questionnaire (GQ)^35^ which is a 21-item that was developed to define the presence of chronic constipation. Only 13 of the 21 items are related to bowel habits. The Rome 111 criteria were used to define constipation. The items are scored using two different four-point Likert scales. The first consisted of ‘never’, ‘sometimes’ (25% of the time) and ‘always’. The second consisted of ‘never’, ‘fewer than once a week’, ‘one or more times a week’ and ‘every day’. Total and cut off scores were not reported.  **Quality Of Life Assessment**  Baseline Quality of life patients will be assess using self-administered PDQ-39^36^ Questionnaire(Malay version) and end of intervention, subject will be assessed again using the same questionnaire. PDQ-39 questionnaire contains 39 items with each item was rated by the patients using one of five categories, from 0 (never) to 4 (always). PDQ-39 summary index (PDQSI) is calculated by dividing the sum of the total raw score by the maximum possible score (156 or 152 points, depending on the patient’s marriage status) and multiplying by 100. In this study, we will use PDQSI as a standardized index for representing PD patients’ life quality.  **Non Motor symptoms (NMS) assessment**  Severity of Non Motor symptoms of each individual will be assess using NMSS Questionnaire. Frequency and severity of each NMS are taken into account. Final total score of disability (0=no disability; 360=maximal disability).  **DDisease severity assessment**  Parkinson Disease severity assessment will be assess using MDS-UPDRS questionnaire. MDS UPDRS has four parts which include nonmotor experiences of daily living (13 items), motor experiences of daily living (13 items), motor examination (18 items), and motor complications (six items). Each subscale now has 0-4 ratings, where 0 = normal, 1 = slight, 2 = mild, 3 = moderate, and 4 = severe.  10.4 **VALIDITY OF TOOLS**  NMS and MDS-UPDRS are public domain questionnaire  Validated score : PDQ 39 (malay version)- permission obtained from author  Garrigues Constipation Questionnaire- awaiting permission from author  **11. STASTICAL ANALYSIS**  Statistical Package for the Social Sciences (SPSS) Software version 2 will be used for statistical analysis. Age, height, weight, body mass index (BMI) and gender were compared between the groups by Student’s t-test and the chi-squared test. General Linear Model will be used to compare intestinal transit time in probiotics and placebo groups before and after 8 weeks of intervention. Pre- and post-probiotics consumption data on clinical evaluation of constipation that will be obtained from all the questionnaires namely Rome III criteria, PDQ-39 Questionnaire and Garrigues Constipation Questionnaire will be analysed using Mc Nemar Test. Independent t-test will be used to compare the differences of intestinal transit time in both groups. Pearson correlation and Simple linear regression analysis will be utilized to explore the relationship and effect between two numerical variables. A p-value <0.05 will be considered as statistically significant.  **11.1 SAMPLE SIZE CALCULATION**  The sample size is being calculated based formula comparing between two proportions. Sample size calculation was performed according to a study by Sakai et al^32^ based on value reported on changes in percentage of bowel movements and complete bowel movements after 3 weeks of probiotics treatment where there was a significant decreased from 73.7% to 36.8%,  Formula for sample size comparing two proportions:  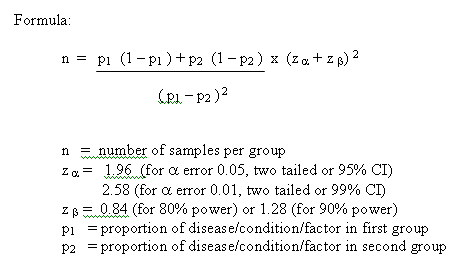  Sample size was estimated as follows :  p1 = 0.737  p2 = 0.368  zα = 1.96  zβ = 0.84  n = 0.737 (1 – 0.737) + 0.368 (1 – 0.368 ) X (1.96 + 0.84) ^2^  (0.737 – 0.368) ^2^  n = 0.193831 + 0.232576 X 7.84  0.136161  n = 25 per group  The number of samples per group is further added 20% to compensate for non-response and lost to follow up. The final samples required for each group is 30 patients. |
| --- | --- | --- | --- | --- | --- | --- | --- | --- | --- | --- | --- | --- | --- | --- | --- |
|  | **12. Study Flow Chart**  **1^ST^ VISIT- WEEK 0**  Patients (n= 60)  Constipation assessment with GQ  Quality of life assessment with PDQ39  Disease severity assessment with MD S- UPDRS  Non motor symptoms assessment with NMS  Probiotics (n=30)  Placebo (n=30)  RE-ASSESSMENT POST INTERVENTION |

**Screening :**

**Rome III Criteria – Constipation Module**

**Negative Fecal occult blood**

**Fulfil inclusion/ exclusion criteria**

2^ND^ VISIT- WEEK 8

Constipation assessment with GQ

Quality of life assessment with PDQ39

Disease severity assessment with MD S- UPDRS

Non motor symptoms assessment with NMS

Questionnaires

Determination GTT

**13. ETHICAL ISSUES**

There will be no ethical issues on this study as following apply:

Consent from patient who agreed to be enrolled for the study of probiotics

Patients were actively monitored for gastrointestinal disorders (common adverse events)

**14. WORKING SCHEDULE**

**14.1 GANTT CHART**

| 2018 | June | July | August | Sept | Oct | Nov | Dec | Jan | Feb | March |
| --- | --- | --- | --- | --- | --- | --- | --- | --- | --- | --- |
|  |  |  |  |  |  |  |  |  |  |  |
| Ethics application and approval |  |  |  |  |  |  |  |  |  |  |
| Patient recruitment and administration of  probiotics |  |  |  |  |  |  |  |  |  |  |
| Sample (faecal), ITT  faecal, clinical data collection and follow-up |  |  |  |  |  |  |  |  |  |  |
| Data analysis and report writing |  |  |  |  |  |  |  |  |  |  |
| Manuscript writing and publication |  |  |  |  |  |  |  |  |  |  |

**15. ESTIMATION OF COST**

| **ITEMS** | | | **COST (RM)** |
| --- | --- | --- | --- |
|  |  |  |  |
|  |  | **Analysis** |  |
|  | i. | Fecal occult blood test (120X RM 15 ) | 1,800.00 |
|  | ii | Capsule Carmine (120X RM 5 ) | 600.00 |
|  | iii | Probiotics | sponsored |
|  | | |  |
| **Total expenses** | | |  |

**REFERRENCE**

1. Barichella M, Cereda E, Pezzoli G. Major nutritional issues in the management of Parkinson’s disease. MovDisord 2009; 24:1881–1892.

2. Fasano A, Visanji NP, Liu LW, Lang AE, Pfeiffer RF. Gastrointestinal dysfunction in Parkinson’s disease. Lancet Neurol 2015; 14:625–639.

3. Berg D, Postuma RB, Adler CH, et al. MDS research criteria for prodromal Parkinson’s disease. MovDisord 2015; 30:1600–1611.

4. Abbott RD, Ross GW, White LR, Sanderson WT, Burchfiel CM, Kashon M, et al. Environmental, life-style, and physical precursors of clinical Parkinson's disease: Recent findings from the Honolulu-Asia Aging Study. J Neurol. 2003; 250:III30–9.

5. C. Singaram, W.Ashraf, E.A.Gaumnitz et al.,“Dopaminergic defect of enteric nervous system in Parkinson’s Disease patients with chronic constipation, “The Lancet,vol.346,no.8979,pp.861–864,1995.

6. Azmin S, Khairul Anuar AM, Tan HJ, et al. Nonmotor Symptoms in a Malaysian Parkinson’s Disease Population. Parkinson’s disease. 2014; 2014:472157.

7. Johanson JF, Kralstein J. Chronic constipation: a survey of the patient perspective. Aliment Pharmacol Ther 2007; 25:599–608.

8. Mu ¨ller-Lissner SA, Kaatz V, Brandt W, Keller J, Layer P. The perceived effect of various foods and beverages on stool consistency. Eur J Gastroenterol Hepatol 2005; 17:109–12.

9. Forsyth CB, Shannon KM, Kordower JH, et al. Increased intestinal permeability correlates with sigmoid mucosa alpha-synuclein staining and endotoxin exposure markers in early Parkinson’s disease. PLoS One 2011; 6:e28032.

10. Keshavarzian A, Green SJ, Engen PA, et al. Colonic bacterial composition in Parkinson’s disease. MovDisord 2015; 30:1351–1360.

11. Hamer HM, Jonkers D, Venema K, Vanhoutvin S, Troost FJ, Brummer RJ. Review article: the role of butyrate on colonic function. Aliment PharmacolTher 2008; 27:104-119.

12. Canani RB, Costanzo MD, Leone L, Pedata M, Meli R, Calignano A. Potential beneficial effects of butyrate in intestinal and extraintestinal diseases. World J Gastroenterol 2011; 17:1519-1528.

13. M.M. Unger, et al., Short chain fatty acids and gut microbiota differ between patients with Parkinson's disease and age-matched controls, Parkinsonism and Related Disorders (2016).

14. C.B. Forsyth, K.M. Shannon, J.H. Kordower, R.M. Voigt, M. Shaikh, J.A. Jaglin, J.D. Estes, H.B. Dodiya, A. Keshavarzian, Increased intestinal permeability correlates with sigmoid mucosa alpha-synuclein staining and endotoxin exposure markers in early Parkinson's disease, PLoS One 6 (12) (2011) e28032.

15. VanZanten, G.C.; Knudsen, A.; R6yti6, H.; Forssten, S.; Lawther, M.; Blennow, A.; Lahtinen, S.J.; Jakobsen, M.; Svensson, B.; Jespersen, L. The effect of selected synbiotics on microbial composition and short chain fatty acid production in a model system of the human colon.PLoS One.2012, 7(10).

16. Noverr, M.C.; Huffuagle G.B. Rationale of Candida albicans morphogenesis by fatty acid metabolites. Infection and immunity. 2004, 72, 6206-6210.

17. Duncan, S. H.; Louis, P .; Flint, H. J. Lactate-Utilizing Bacteria, Isolated from Human Feces, That Produce Butyrate as a Major Fermentation Product. ApplEnvironMicrobiol. 2004, 70(10): 5810-5817.

18. Bourriaud, C., S. Akoka, S. Goupry, R. Robins, C. Cherbut, and C. Michel. 2002. Butyrate production from lactate by human colonic microflora. Reprod. Nutr. Dev. 42(Suppl. 1):S55.

19. Food and Agriculture Organization of the United Nations and WHO Working Group. Guidelines for the evaluation of probiotics in food. Geneva, Switzerland: FAO/WHO, 2002.

20. Chmielewska A, Szajewska H. Systematic review of randomised controlled trials: probiotics for functional constipation. World J Gastroenterol 2010; 16:69–75.

21. Khalif IL, Quigley EM, Konovitch EA, Maximova ID. Alterations in the colonic ﬂora and intestinal permeability and evidence of immune activation in chronic constipation. Dig Liver Dis 2005; 37:838–49.

22. Zoppi G, Cinquetti M, Luciano A, Benini A, Muner A, Bertazzoni Minelli E. The intestinal ecosystem in chronic functional constipation. Acta Paediatr 1998; 87:836–41.

23. 16. Ait-Belgnaoui A, Han W, Lamine F, Eutamene H, Fioramonti J, Bueno L, Theodorou V. Lactobacillus farciminis treatment suppresses stress induced visceral hypersensitivity: a possible action through interaction with epithelial cell cytoskeleton contraction. Gut 2006; 55:1090–4.

24. Rousseaux C, Thuru X, Gelot A, Barnich N, Neut C, Dubuquoy L, Dubuquoy C, Merour E, Geboes K, Chamaillard M, et al. Lactobacillus acidophilus modulates intestinal pain and induces opioid and cannabinoid receptors. Nat Med 2007; 13:35–7.

25. Bueno L, de Ponti F, Fried M, Kullak-Ublick GA, Kwiatek MA, Pohl D, Quigley EM, Tack J, Talley NJ. Serotonergic and non-serotonergic targets in the pharmacotherapy of visceral hypersensitivity. Neurogastroenterol Motil 2007; 19(suppl):89–119.

26. Quigley EM. Bacteria: a new player in gastrointestinal motility disorders– infections, bacterial overgrowth, and probiotics. Gastroenterol Clin North Am 2007; 36:735–48.

27. Waller PA, Gopal PK, Leyer GJ, Ouwehand AC, Reifer C, Stewart ME, Miller LE. Dose-response effect of Biﬁdobacterium lactis HN019 on whole gut transit time and functional gastrointestinal symptoms in adults. Scand J Gastroenterol 2011; 46:1057–64.

28. Salminen S, Salminen E. Lactulose, lactic acid bacteria, intestinal microecologyandmucosalprotection.ScandJ GastroenterolSuppl1997;222:45–8.

29.Michela Barichella, Claudio Pacchetti, Carlotta Bolliri, Erica Cassani, Laura Iorio, Chiara Pusani, Giovanna Pinelli, Giulia Privitera, IlariaCesari, Samanta Andrea Faierman, Riccardo Caccialanza, GianniPezzoli, Emanuele Cereda . Neurology Sep 2016, 87 (12) 1274-1280;

30. Chapman CM, Gibson GR, Rowland I. Health benefits of probiotics: are mixtures more effective than single strains? Eur J Nutr 2011; 50:1–17.

31. Dimidi E, Christodoulides S, Fragkos KC, Scott SM, Whelan K. The effect of probiotics on functional constipation in adults: a systematic review and meta-analysis of randomized controlled trials. Am J ClinNutr 2014; 100: 1075– 1084.

32. Sakai, T., Makino, H., Ishikawa, E., Oishi, K. and Kushiro, A. (2011) Fermented milk containing Lactobacillus casei strain Shirota reduces incidence of hard or lumpy stools in healthy population. International Journal of Food Sciences and Nutrition

33. Drossman DA. The functional gastrointestinal disorders and the Rome III process. Gastroenterology 2006; 130: 1377–1390.

35. Garrigues V, Ga ´lvez C, Ortiz V, Ponce M, Nos P, Ponce J. 2004. Prevalence of constipation: Agreement among several criteria and evaluation of the diagnostic accuracy of qualifying symptoms and self-reported deﬁnition in a population-based survey in Spain. Am J Epidemiol 159:520–526

36. Peto v, Jenkinson C, Fitzpatrick R. PDQ-39: a review of the development, validation and application of a Parkinson's disease quality of life questionnaire and its associated measures. Journal of Neurology, 1998; 245 (Suppl.1):S10-S14

37. Dupont WD, Plummer WD: "Power and Sample Size Calculations: A Review and Computer Program", Controlled Clinical Trials 1990; 11:116-28.

| **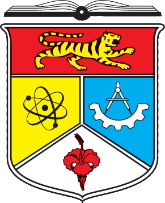** | **THE EFFECTIVENESS OF PROBIOTICS**  **IN PARKINSON DISEASE WITH CONSTIPATION**  **(SCREENING FORM)** | **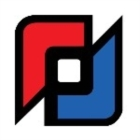** |
| --- | --- | --- |

(Sticker)

Name :

IC No :

UKM MRN No :

Sex : Male / Female

Age :

Race : Malay / Chinese / Indian / Others

Religion : Islam / Buddhist / Hindu / Christian / Others

Body weight :

Height :

BMI :

Duration of Parkinson Disease:

Disease severity (MDS - UPDRS):

Fulfill Rome III Criteria – Constipation : YES/NO

|  | | | | | | |
| --- | --- | --- | --- | --- | --- | --- |
| Indicated the amount of fibre in your diet: | | | | |  | |
|  | Low (less than 3 servings per day) | | | |  | |
|  | Medium (3-5 servings per day) | | | |  | |
|  | High (more than 5 servings per day) | | | | *1 serving = ½ cup | |
|  |  | | | |  | |
| How often do you perform physical exercise? | | | | |  | |
|  | |  |  |  |  |  |
|  | Sedentary (less than 4 hour per week) | | | |  | |
|  | Non Sedentary (more than 4 hour per week) | | | |  | |
|  | | | | |  | |
| Current medications: | | |  | Previous colonoscopy(within 5 years) | | |
|  | | |  | Year | | Diagnosis |

| Fecal occult blood screening |
| --- |
|  |

| Name:  MRN:  (Sticker) |  | Day 0 |  |  |
| --- | --- | --- | --- | --- |
|  |  | Date | / / 2018 | |

**Rome III Criteria – Constipation Module^1^ (CONSTIPATION SCREENING FORM)**

| **Q1.** In the last 3 months, how often did you have discomfort or pain anywhere in your abdomen? | | |  | **Q11.** In the last 3 months, how often did you strain during bowel movements? | | |
| --- | --- | --- | --- | --- | --- | --- |
|  | Never |  |  |  | Never or rarely |  |
|  | Less than one day a month |  |  |  | Sometimes |  |
|  | One day a month |  |  |  | Often |  |
|  | Two to three days a month |  |  |  | Most of the time |  |
|  | One day a week |  |  |  | Always |  |
|  | More than one day a week |  |  |  |  |  |
|  | Every day |  |  |  |  |  |
|  | | |  |  |  |  |
| **Q2.** For women: Did this discomfort or pain occur only during your menstrual bleeding and not at other times? | | |  | **Q12.** In the last 3 months, how often did you have a feeling of incomplete emptying after the bowel movements? | | |
|  | No |  |  |  | Never or rarely |  |
|  | Yes |  |  |  | Sometimes |  |
|  | Does not apply because I have had the change in life (menopause) or I am a male |  |  |  | Often |  |
|  |  |  |  |  | Most of the time |  |
|  |  |  |  |  | Always |  |
|  | | |  |  |  |  |

| **Q3.** Have you had this discomfort or pain 6 months or longer? | | | |  | **Q13.** In the last 3 months, how often did you have a sensation that the stool could not be passed, (i.e. , blocked), when having a bowel movement? | | |
| --- | --- | --- | --- | --- | --- | --- | --- |
|  | No | |  |  |  | Never or rarely |  |
|  | Yes | |  |  |  | Sometimes |  |
|  |  | |  |  |  | Often |  |
|  |  | |  |  |  | Most of the time |  |
|  |  | |  |  |  | Always |  |
|  | | | |  |  |  |  |
| **Q4.** How often did this discomfort or pain get better or stop after you had a bowel movement? | | | |  | **Q14.** In the last 3 months, how often did you press on or around your bottom or remove stool in order to complete a bowel movement? | | |
|  | Never or rarely | |  |  |  | Never or rarely |  |
|  | Sometimes | |  |  |  | Sometimes |  |
|  | Often | |  |  |  | Often |  |
|  | Most of the time | |  |  |  | Most of the time |  |
|  | Always | |  |  |  | Always |  |
|  | | | |  |  |  |  |
| **Q5.** When this discomfort or pain started, did you have more frequent bowel movements? | | | |  | **Q15.** In the last 3 months, how often did you have difficulty relaxing or letting go to allow the stool to come out during a bowel movement? | | |
|  | Never or rarely | |  |  |  | Never or rarely |  |
|  | Sometimes | |  |  |  | Sometimes |  |
|  | Often | |  |  |  | Often |  |
|  | Most of the time | |  |  |  | Most of the time |  |
|  | Always | |  |  |  | Always |  |
|  |  | |  |  |  |  |  |
| **Q6.**  When this discomfort or pain started, did you have less frequent bowel movements? | | | |  | **Q16.** Did any of the symptoms of constipation listed in question 9-15 above begin more than 6 months ago? | | |
|  | | Never or rarely | |  |  | Yes |  |
|  | | Sometimes | |  |  | No |  |
|  | | Often | |  |  |  |  |
|  | | Most of the time | |  |  |  |  |
|  | | Always | |  |  |  |  |
|  | | | |  |  |  |  |
| **Q7.** When this discomfort or pain started, were your stools (bowel movements) looser? | | | |  | **Q17.** In the last 3 months, how often did you have loose mushy or watery stool? | | |
|  | | Never or rarely | |  |  | Never or rarely |  |
|  | | Sometimes | |  |  | Sometimes |  |
|  | | Often | |  |  | Often |  |
|  | | Most of the time | |  |  | Most of the time |  |
|  | | Always | |  |  | Always |  |
|  | | | |  |  |  |  |
|  | | | |  |  |  |  |

| **Q8.** When this discomfort or pain started, how often did you have harder stools? | |
| --- | --- |
|  | Never or rarely |
|  | Sometimes |
|  | Often |
|  | Most of the time |
|  | Always |
|  | |
| **Q9.** In the last 3 months, how often did you have fewer than three bowel movements (0-2) a week? | |
|  | Never or rarely |
|  | Sometimes |
|  | Often |
|  | Most of the time |
|  | Always |
|  | |
| **Q10.** In the last 3 months, how often did you have hard or lumpy stools? | |
|  | Never or rarely |
|  | Sometimes |
|  | Often |
|  | Most of the time |
|  | Always |

^1^Drossman et al *Rome III The Functional Gastrointestinal Disorders (3^rd^ Edition)*, August 2006

| Name:  MRN:  (Sticker) |  | Day 0 |  |  |
| --- | --- | --- | --- | --- |
|  |  | Date | / / 2018 | |

**Garrigues Survey – Constipation^1^**

|  |  | | | |  | |  | |  |  |  |
| --- | --- | --- | --- | --- | --- | --- | --- | --- | --- | --- | --- |
| **Q1.** Indicate your age. | | | | | | |  | | **Q11.** How often are your stools hard? | | |
|  | | | | |  | |  | |  | Never |  |
|  |  |  |  |  |  | |  | |  | Sometimes (less than 25% of time) |  |
|  |  |  |  |  |  | |  | |  | Often (more than 25% of time) |  |
|  |  |  |  |  |  | |  | |  | Always |  |
|  | | | | | | |  | |  |  |  |
| **Q2.** Indicate your gender. | | | | | | |  | | **Q12.** Do you feel a blockage in the anus that makes it difficult to pass the stool? | | |
|  | Male | | | |  | |  | |  | Never |  |
|  | Female | | | |  | |  | |  | Sometimes (less than 25% of time) |  |
|  |  | | | |  | |  | |  | Often (more than 25% of time) |  |
|  |  |  |  |  |  |  |  |  |  | Always |  |
|  |  | | | |  | |  | |  |  |  |
| **Q3.** Indicate your educational level | | | | | | |  | | **Q13.** Do you need to press around the anus or vagina to complete a bowel movement? | | |
|  | Primary | | | |  | |  | |  | Never |  |
|  | Secondary | | | |  | |  | |  | Sometimes (less than 25% of time) |  |
|  | Tertiary | | | |  | |  | |  | Often (more than 25% of time) |  |
|  | Postgraduate | | | |  | |  | |  | Always |  |
|  |  | | | |  | |  | |  |  |  |
| **Q4.** Indicate your job | | | | | | |  | | **Q14.** Do you spend more than 10 minutes on the toilet to pass the stools? | | |
|  | | | | | | |  | |  | Never |  |
|  |  |  |  |  |  |  |  | |  | Sometimes (less than 25% of time) |  |
|  |  |  |  |  |  |  |  | |  | Often (more than 25% of time) |  |
|  |  |  |  |  |  |  |  | |  | Always |  |
|  | | | | | | |  | |  |  |  |
| **Q5.** Indicate the amount of fibre in your diet | | | | | | |  | | **Q15.** How many bowel movements do you usually have each week? | | |
|  | Low (less than 3 servings per day) | | | |  | |  | |  | | |
|  | Medium (3-5 servings per day) | | | |  | |  | |  |  |  |
|  | High (more than 5 servings per day) | | | |  | |  | |  |  |  |
|  | *1 serving = ½ cup | | | |  | |  | |  |  |  |
| **Q6.**  How often do you perform physical exercise? | | | | | | |  | | **Q16.** Do you take oral laxatives? | | |
|  | | Never | | | | |  | |  | Never |  |
|  | | Sometimes (less than 3 times per week) | | | | |  | |  | Fewer than once a week |  |
|  | | Habitually (more than 3 times per week) | | | | |  | |  | One or more times a week |  |
|  | |  | | | | |  | |  | Every day |  |
|  | | | | | | |  | |  |  |  |
| **Q7.** Indicate which drugs you are taking | | | | | | |  | | **Q17.** Do you need to use suppositories to have bowel movements? | | |
|  | | Non steroidal anti inflammatory (NSAIDs) | | | | |  | |  | Never |  |
|  | | Anxiolytics | | | | |  | |  | Fewer than once a week |  |
|  | | Calcium channel antagonist | | | | |  | |  | One or more times a week |  |
|  | | Female hormone based drugs | | | | |  | |  | Every day |  |
|  | | |  |  | |  | |  |  |  |  |
|  | | | | | | |  | |  |  |  |
| **Q8.** Have you felt constipated? | | | | | | |  | | **Q18.** Do you need to use enemas to have bowel movements? | |  |
|  | | Yes | | | | |  | |  | Never |  |
|  | | No | | | | |  | |  | Fewer than once a week |  |
|  | |  | | | | |  | |  | One or more times a week |  |
|  | |  | | | | |  | |  | Every day |  |
|  | |  | | | | |  | |  |  |  |
| **Q9.** Do you strain during a bowel movement? | | | | | | |  | | **Q19.** Have you visited a doctor because of constipation? | |  |
|  | | Never | | | | |  | |  | Yes |  |
|  | | Sometimes (less than 25% of time) | | | | |  | |  | No |  |
|  | | Often (more than 25% of time) | | | | |  | |  |  |  |
|  | | Always | | | | |  | |  | |  |
|  | |  | | | | |  | |  |  |  |
| **Q10.** Do you feel an incomplete emptying sensation after a bowel movement? | | | | | | |  | | **Q20.** Have you presented with abdominal pain more than six times this past year? | |  |
|  | | Never | | | | |  | |  | Yes |  |
|  | | Sometimes (less than 25% of time) | | | | |  | |  | No |  |
|  | | Often (more than 25% of time) | | | | |  | |  |  |  |
|  | | Always | | | | |  | |  |  |  |
|  | |  | | | | |  | |  | |  |
|  | |  | | | | |  | | **Q21.** Have you presented with loose or watery stools? | |  |
|  | |  | | | | |  | |  | Yes |  |
|  | |  | | | | |  | |  | No |  |
|  | |  | | | | |  | |  |  |  |

^1^Garrigues et al *Am J Epidemiol* 2004

PDQ-39 (MALAY)

| Name:  MRN:  (Sticker) |  | Day 0 |  |  |
| --- | --- | --- | --- | --- |
|  |  | Date | / / 2018 | |

# **Penyakit Parkinson Soal Selidik Mutu Hidup (PDQ-39)**

Disebabkan menghidap penyakit Parkinson,
berapa kerapkah dalam sebulan yang lalu telah anda…

Sila **tandakan satu kotak** bagi setiap soalan

|  |  |  | Tidak Pernah | Jarang-jarang sekali | Kadang-kadang | Sering | Selalu  atau tidak boleh lakukan langsung |
| --- | --- | --- | --- | --- | --- | --- | --- |
| 1. | Menghadapi kesukaran melakukan kegiatan riadah (kegiatan waktu lapang) yang ingin anda lakukan? |  |  |  |  |  |  |
| 2. | Menghadapi kesukaran menjaga rumah anda seperti kerja bertukang sendiri (tugas-tugas minor di dalam rumah seperti membina rak atau membaiki almari),  kerja rumah, memasak? |  |  |  |  |  |  |
| 3. | Menghadapi kesukaran mengangkat  beg-beg perbelanjaan harian? |  |  |  |  |  |  |
| 4. | Menghadapi masalah berjalan sejauh setengah batu (kira-kira 800 meter)? |  |  |  |  |  |  |
| 5. | Menghadapi masalah berjalan sejauh 100 ela (kira-kira 90 meter)? |  |  |  |  |  |  |
| 6. | Menghadapi masalah pergerakan  di dalam rumah semudah yang  anda inginkan? |  |  |  |  |  |  |

Sila periksa bahawa anda telah menandakan satu kotak bagi setiap soalan
sebelum meneruskan dengan muka surat yang berikutnya.

**Disebabkan menghidap penyakit Parkinson,**berapa kerapkah dalam sebulan yang lalu telah anda…

Sila **tandakan satu kotak** bagi setiap soalan

|  |  |  | Tidak Pernah | Jarang-jarang sekali | Kadang-kadang | Sering | Selalu  atau tidak boleh lakukan langsung |
| --- | --- | --- | --- | --- | --- | --- | --- |
| 7. | Menghadapi kesukaran berjalan-jalan  di tempat awam? |  |  |  |  |  |  |
| 8. | Memerlukan seseorang untuk menemani anda semasa anda keluar? |  |  |  |  |  |  |
| 9. | Berasa takut atau bimbang tentang kemungkinan anda tersungkur atau terjatuh di tempat awam? |  |  |  |  |  |  |
| 10. | Terkurung di dalam rumah lebih dari  apa yang anda inginkan? |  |  |  |  |  |  |
| 11. | Menghadapi kesukaran membersihkan diri anda? |  |  |  |  |  |  |
| 12. | Menghadapi kesukaran memakai  pakaian sendiri? |  |  |  |  |  |  |
| 13. | Menghadapi masalah membutangkan pakaian anda atau mengikat tali kasut? |  |  |  |  |  |  |
| 14. | Menghadapi masalah untuk menulis dengan jelas? |  |  |  |  |  |  |

Sila periksa bahawa anda telah menandakan satu kotak bagi setiap soalan
sebelum meneruskan dengan muka surat yang berikutnya

Disebabkan menghidap penyakit Parkinson,
berapa kerapkah dalam sebulan yang lalu telah anda…

Sila **tandakan satu kotak** bagi setiap soalan

|  |  |  | Tidak Pernah | Jarang-jarang sekali | Kadang-kadang | Sering | Selalu  atau tidak boleh lakukan langsung |
| --- | --- | --- | --- | --- | --- | --- | --- |
| 15. | Menghadapi masalah ketika menggunakan garpu dan pisau untuk memotong makanan anda? |  |  |  |  |  |  |
| 16. | Menghadapi kesukaran memegang  minuman tanpa sebarang tumpahan? |  |  |  |  |  |  |
| 17. | Berasa muram? |  |  |  |  |  |  |
| 18. | Berasa tersisih dan kesunyian? |  |  |  |  |  |  |
| 19. | Berasa mudah menangis atau hendak menangis? |  |  |  |  |  |  |
| 20. | Berasa marah atau berkecil hati? |  |  |  |  |  |  |
| 21. | Berasa cemas? |  |  |  |  |  |  |
| 22. | Berasa bimbang tentang masa hadapan anda? |  |  |  |  |  |  |
| 23. | Berasa anda terpaksa menyembunyikan penyakit Parkinson yang anda hadapi dari pengetahuan orang sekeliling anda? |  |  |  |  |  |  |

Disebabkan menghidap penyakit Parkinson,
berapa kerapkah dalam sebulan yang lalu telah anda…

Sila **tandakan satu kotak** bagi setiap soalan

|  |  |  | Tidak Pernah | Jarang-jarang sekali | Kadang-kadang | Sering | Selalu |
| --- | --- | --- | --- | --- | --- | --- | --- |
| 24. | Menjauhkan diri daripada makan atau  minum di tempat awam? |  |  |  |  |  |  |
| 25. | Berasa malu di tempat awam kerana menghidapi penyakit Parkinson? |  |  |  |  |  |  |
| 26. | Berasa bimbang akan tanggapan (reaksi) orang lain terhadap diri anda? |  |  |  |  |  |  |
| 27. | Menghadapi masalah dengan perhubungan rapat dan peribadi anda? |  |  |  |  |  |  |
| 28. | Adakah anda kekurangan sokongan daripada suami/isteri atau teman hidup anda? *Jika anda tidak mempunyai suami/isteri  atau teman hidup, sila tandakan di sini* |  |  |  |  |  |  |
| 29. | Adakah anda kekurangan sokongan daripada keluarga atau kawan-kawan rapat anda? |  |  |  |  |  |  |
| 30. | Tanpa disangka-sangka, tertidur di siang hari? |  |  |  |  |  |  |
| 31. | Menghadapi masalah dengan tumpuan anda, misalnya semasa membaca atau menonton TV? |  |  |  |  |  |  |

Disebabkan menghidap penyakit Parkinson,
berapa kerapkah dalam sebulan yang lalu telah anda…

Sila **tandakan satu kotak** bagi setiap soalan

|  |  |  | Tidak Pernah | Jarang-jarang sekali | Kadang-kadang | Sering | Selalu |
| --- | --- | --- | --- | --- | --- | --- | --- |
| 32. | Merasakan bahawa daya ingatan anda kurang baik? |  |  |  |  |  |  |
| 33. | Mengalami mimpi yang mencemaskan  atau mengalami khayalan/bayangan? |  |  |  |  |  |  |
| 34. | Mempunyai kerumitan dengan pertuturan anda? |  |  |  |  |  |  |
| 35. | Berasa tidak boleh berhubung dengan  orang dengan sempurna? |  |  |  |  |  |  |
| 36. | Berasa tidak diendahkan oleh orang lain? |  |  |  |  |  |  |
| 37. | Mengalami kesakitan akibat kekejangan  atau spasma (pengecutan otot luar kawal)? |  |  |  |  |  |  |
| 38. | Mengalami kesakitan dan rasa lenguh  pada sendi atau tubuh? |  |  |  |  |  |  |
| 39. | Berasa kurang selesa kerana kepanasan atau kesejukan? |  |  |  |  |  |  |

Sila periksa bahawa anda telah menandakan satu kotak bagi setiap soalan.


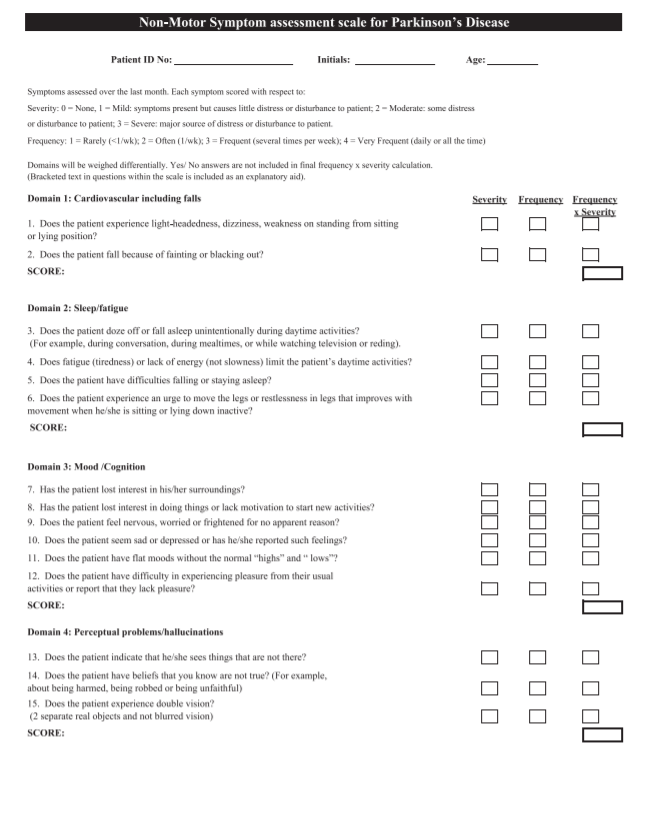


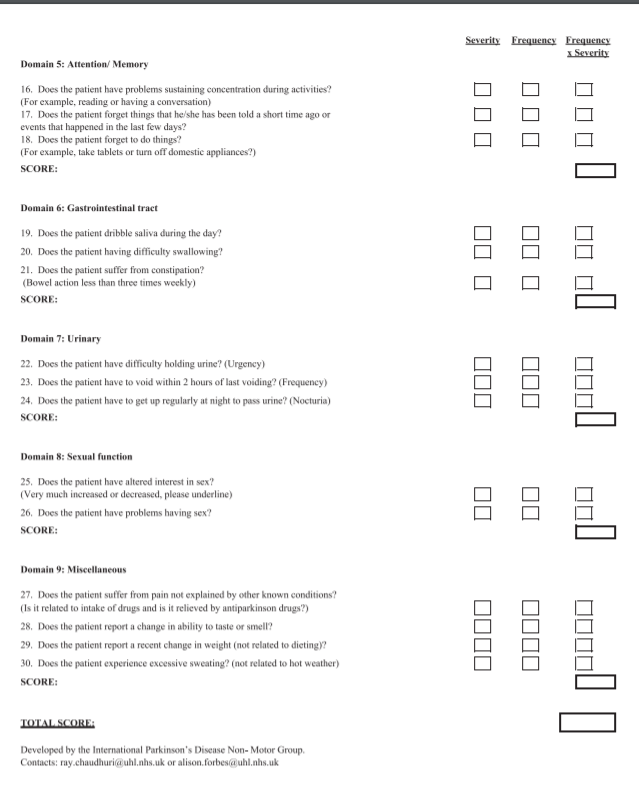


| Name:  MRN:  (Sticker) |  | Day 0 |  |  |
| --- | --- | --- | --- | --- |
|  |  | Date | / / 2018 | |


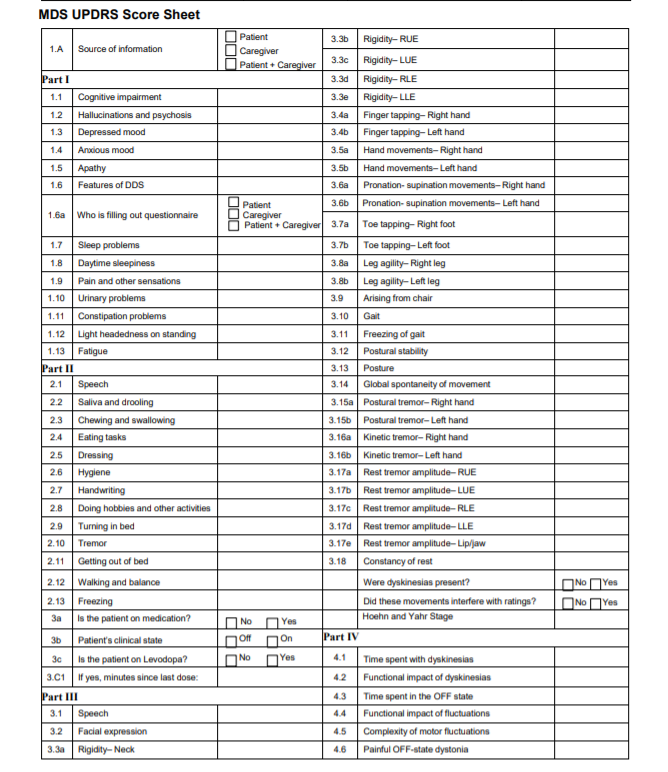


**healthoutcomes@innovation.ox.ac.uk**

**To:**maizarah_84@yahoo.com

Jul 2 at 11:06 PM

**Order PDQ-3-110636 has been approved**

Dear AZLIZA IBRAHIM

I am pleased to inform you that your request to use the PRO measure was successful and you now have a licence To use it.

[You can download your documents here](https://process.innovation.ox.ac.uk/clinical/Download/7c98f125-2364-4252-a740-cc8596893cb6)

If you have any further questions please contact Clinical Outcomes at [healthoutcomes@innovation.ox.ac.uk](mailto:healthoutcomes@innovation.ox.ac.uk)

Regards

Clinical Outcomes at Oxford University Innovation

Informed Consent Sheet (Malay)
MAKLUMAT UNTUK PESERTA

Tajuk Penyelidikan

Penilaian Kesan Minuman Susu Kultur Ke Atas Pesakit Parkinson Yang Mengalami Sembelit.

Pengenalan

Anda dijemput untuk mengambil bahagian dalam kajian penyelidikan ini. Sebelum mengambil bahagian, adalah penting bagi anda mengambil masa untuk membaca dan memahami kandungan di dalam helaian maklumat ini.

Tujuan Penyelidikan

Sembelit ialah keadaan perubatan yang sering dilaporkan di klinik penjagaan utama dikalangan pesakit Parkinson. Salah satu punca utama masalah sembelit ialah pergerakan usus yang lambat dan masalah berkaitan saraf pada pesakit Parkinson. Pengambilan produk minuman susu kultur telah dilaporkan dapat membantu mengatasi masalah ini. Pengambilan secara berterusan boleh meningkatkan pergerakan usus serta boleh membawa kebaikan dan membantu masalah sembelit. Tujuan penyelidikan ini ialah untuk menentukan keberkesanan minuman susu kultur selama 8 minggu pengambilan ke atas pesakit parkinson yang mengalami sembelit.

Apa yang akan dilakukan?

Anda akan disoal dengan beberapa soalan bagi melengkapkan borang soal-selidik demographic yang merangkumi perincian tentang nama,umur, jantina, bangsa dan agama, sejarah perubatan, latar belakang ekonomi, diet dan aktiviti fizikal. Selepas itu, anda akan di bahagi kepada dua kumpulan yang akan menerima susu kultur atau plasebo selama 8 minggu dan perlu di ambil dua kali sehari. Sebelum anda di bekalkan dengan susu kultur atau placebo ini , anda diminta untuk melakukan ujian ‘fecal occult blood ’. Ujian ini merangkumi pengambilan spesimen najis yang akan di hantar ke makmal HUKM bagi tujuan ujian darah di dalam najis.Jika ujian ‘fecal occult blood test’ ini positive, anda akan digugurkan dari menyertai penyelidikan ini dan seterusnya anda akan di rujuk ke pakar usus bagi ujian endoscopic (colonoscopy) dan rawatan selanjutnya.

Selain itu, anda juga akan di soal selidik melalui borang selidik seperti berikut pada temu janji pertama :

1) Garrigues Constipation questionnaire – soal selidik simptom sembelit

2) PDQ39 questionnaire – soal selidik mutu hidup Penyakit Parkinson (pesakit mengisi sendiri borang soal selidik ini)

3) NMSS questionnaire- soal selidik simptom non motor Parkinson

4) MDS-UPDRS – soal selidik dan pemeriksaan clinical mengenai keterukan penyakit Parkinson disease.

Selepas itu, anda akan di bekalkan minuman susu kultur selama 8 minggu dan perlu di ambil selama dua kali sehari. Sebelum anda mengambil minuman susu kultur ini, anda juga akan di beri sejenis ubat yang bernama ‘ capsule carmine’. Tujuan pemberian ubat ini adalah untuk mengukur perubahan jangka masa penghasilan najis sebelum dan selepas minuman susu kultur. Ubat ini tidak mengandungi bahan toksik dan akan memberi warna merah kepada najis, Anda perlu menelan sebanyak 4 biji ubat ini selepas pembuangan najis yang terakhir. Anda perlu merekodkan masa pengambilan ubat ini dan masa penghasilan najis merah yang seterusnya .Ujian ini juga akan di ulangi sekali lagi selepas pesakit menghabiskan minuman susu kultur yang di bekalkan selama 8 minggu.

Selepas pesakit menghabiskan minuman susu kultur selama 8 minggu, pesakit akan juga mengulangi semula ujian soal selidik yang sama seperti yang tertera di atas.

.

Faedah Penyelidikan

Pengambilan bahagian dalam penyelidikan ini tidak memberi faedah terus kepada anda. Minuman susu kultur yang diberi mungkin membantu atau tidak membantu meningkatkan pergerakan usus anda. Namun begitu, penglibatan anda dapat membantu kami membaiki cara untuk mengatasi masalah sembelit untuk pesakit parkinson di masa hadapan.

Risiko

Tiada risiko tambahan

Data dan kerahsiaan

Data yang diperolehi daripada penyelidikan ini akan dihasilkan dalam bentuk laporan yang akan diterbit. Akses kepada data hanya oleh ahli kumpulan penyelidikan ini dan Jawatankuasa Etika Penyelidikan UKM. Data akan dilaporkan secara keseluruhan tanpa merujuk kepada seseorang individu. Oleh yang demikian, identiti anda adalah sulit.

Perlukah saya mengambil bahagian?

Penglibatan anda dalam penyelidikan ini adalah secara sukarela. Sekiranya anda bersetuju untuk mengambil bahagian, anda akan diminta untuk menandatangani “Borang Keizinan”. Anda akan diberi satu salinan borang tersebut dan helaian maklumat ini.

Sekiranya anda tidak bersetuju untuk mengambil bahagian dalam penyelidikan ini, rawatan anda tidak akan terjejas. Anda akan menjalani pemeriksaan di klinik Parkinson seperti biasa.

Anda masih boleh menarik diri daripada penyelidikan ini tanpa sebarang alasan. Data anda tidak akan digunakan dan tidak akan di senaraikan dalam penyelidikan ini lagi.

Siapakah yang boleh saya hubungi mengenai penyelidikan ini?

Sekiranya anda mempunyai sebarang pertanyaan, anda boleh menghubungi ahli kumpulan penyelidikan ini. Anda juga boleh menghubungi penyelidik untuk maklumat lanjut.

Prof. Datin Dr. Norlinah Mohamed Ibrahim

Jabatan Perubatan,

Fakulti Perubatan,

Hospital Canselor Tuanku Muhriz,

Pusat Perubatan UKM (PPUKM),

Jalan Ya’acob Latiff, Bandar Tun Razak,

56000 Cheras, Kuala Lumpur

Tel : 03-91456094

Fax : 03-91456679

Dr Azliza Binti Ibrahim

Jabatan Perubatan,

Fakulti Perubatan,

Hospital Canselor Tuanku Muhriz,

Pusat Perubatan UKM (PPUKM)

Jalan Ya’acob Latiff, Bandar Tun Razak,

56000 Cheras, Kuala Lumpur,

Tel : 0172640678, 03-91456094

Fax : 03-91456679

**BORANG KEIZINAN**

**Tajuk Penyelidikan:**

Penilaian Kesan Minuman Susu Kultur ke atas Pesakit Parkinson yang Mengalami Sembelit.

**Nama Penyelidik:** Prof. Datin Dr. Norlinah Mohamed Ibrahim.

**Persetujuan**

Saya,….………………………………………………………., No Kad Pengenalan: ………………..…………….....

- telah membaca maklumat dalam Helaian Maklumat untuk Peserta **termasuk maklumat tentang risiko dalam penyelidikan ini .**
- telah diberi masa untuk memikirkan tentang penyelidikan ini dan semua pertanyaan daripada saya telah dijawab serta memenuhi kepuasan saya.
- faham bahawa saya bebas untuk menarik diri daripada penyelidikan ini pada bila-bila masa tanpa memberi sebab.
- faham bahawa identiti saya tidak akan didedahkan dalam laporan.

Saya, dengan ini bersetuju secara sukarela untuk mengambil bahagian dalam penyelidikan ini, mengikut prosedur kajian and memberi maklumat yang diperlukan kepada doktor, jururawat atau kakitangan.

Tandatangan : Tarikh:

| **Saksi**  …………………………………………………………….  (Nama)  …………………………………………………………….  (No. Kad Pengenalan)  …………………………………………………………….  (Tandatangan)  …………………………………………………………….  (Tarikh) | **Penyelidik**  …………………………………………………………….  (Nama)  …………………………………………………………….  (No. Kad Pengenalan)  …………………………………………………………….  (Tandatangan)  …………………………………………………………….  (Tarikh) |
| --- | --- |

INFORMATION SHEET FOR PARTICIPANT

Research Title

The effectiveness of Probiotics on Constipation in Parkinson Disease.

Introduction

You are invited to participate in a research study. Before participating in this study, it is important that you take time to read and understand the information in this Information Sheet.

Purpose of Study

Constipation is a medical condition frequently reported in Parkinson Disease patients. One of the major causes for constipation is slow bowel movement and neurological cause. Consumption of probiotics has been shown to improve constipation with regular consumption may improve bowel habits and could be beneficial for patients. The aim of this study is to determine the effect of probiotics consumption for total of 8 weeks in Parkinson Disease patients with constipation. The measurements include duration of gastrointestinal transit time (GTT) which is time of consumption until bowel movement (GTT) and the clinical evaluation of constipation symptom.

What will the study involve?

You will be asked a series of questions to complete a questionnaire which includes information on name, age, sex, race, medical history, background economy details and diet. You will then divided into two groups where you will received either probiotics or placebo. Stool samples will be collected for fecal occult blood testing (testing blood in stool) prior probiotics or placebo consumption. If the fecal occult blood tested positive, you will be withdrawn from this study and will be referred to gastroenterologist for colonoscopy and further assessment.

Apart from that, you also have to answer and fill up questionnaire forms. There will be 4 types of questionnaire forms:

1) Garrigues Constipation questionnaire – to assess constipation symptoms

2) PDQ39 questionnaire –to assess quality of life (self –administered)

3) NMSS questionnaire- to assess non motor symptoms of Parkinson Disease

4) MDS-UPDRS – to assess the severity of Parkinson Disease

You will be asked again on the above questionnaire after completed 8 weeks of probiotics.

You will be provided probiotics for total of 8 weeks to be taken twice daily. Before consumption of this probiotics, you will be instructed to ingest 4 red carmine capsule which is a non-toxic food colorant after the last bowel opening. This carmine capsule will give a red appearance to stool and time from the ingestion of the red carmine capsules and first appearance of red colored stool need to be recorded. Basically, the test is done to measure gastrointestinal transit time (GTT) and it will be repeated after consumption of probiotics for total of 8 weeks to look for GTT improvement.

.

The benefits

There is unlikely to be a direct benefit for you in participating in this study. The probiotics given may or may not help to improve your bowel movement. However, your participation could help us improve on the measures to alleviate constipation condition in future patients.

The risks

There are no additional risks involved.

Data and confidentiality.

The data from this study will be made into a report which may be published. Access to the data is only by the research team and the REC UKM. The data will be reported in a collective manner with no reference to an individual. Hence your identity will be kept confidential.

Do I have to take part?

Participation in this study is voluntary. If you agree to take part, then you will be asked to sign the “Informed Consent Form”. You will be given a copy of the form and this Information Sheet. Your treatment is not affected if you decide not to participate in this study. You will undergo Parkinson clinic follow up as usual. Should you decide to participate, you can still withdraw from the study without penalty. Your data will not be used and will be discarded

Payment

You do have to pay for the usual hospital charges.

This study is being conducted by researchers from UKM Medical Centre.

Who can I ask about the study?

If you have any questions, you can direct them to the research team. You can also contact the researchers for clarifications.

Prof. Datin Dr. Norlinah Mohamed Ibrahim

Jabatan Perubatan,

Fakulti Perubatan,

Hospital Canselor Tuanku Muhriz,

Pusat Perubatan UKM (PPUKM),

Jalan Ya’acob Latiff, Bandar Tun Razak,

56000 Cheras, Kuala Lumpur

Tel : 03-91456094

Fax : 03-91456679

Dr Azliza Binti Ibrahim

Jabatan Perubatan,

Fakulti Perubatan,

Hospital Canselor Tuanku Muhriz,

Pusat Perubatan UKM (PPUKM)

Jalan Ya’acob Latiff, Bandar Tun Razak,

56000 Cheras, Kuala Lumpur,

Tel : 017-2640678, 03-91456094/ Fax : 03-91456679

**INFORMED CONSENT FORM**

**Research Title:** The effectiveness of Probiotics on Constipation in Parkinson Disease

**Researcher’s Name:** Prof. Datin Dr. Norlinah Mohamed Ibrahim

**Consent:**

I, …………………………………………………………………………., IC No: ……………………..………………….....

- have read the information in the Patient Information Sheet **including information regarding the risk in this study**
- Have been given time to think about it and all of my questions have been answered to my satisfaction.
- Understand that I may freely choose to withdraw from this study at any time without given any reason.
- Understand that my anonymity will be ensured in the write-up.

I hereby, voluntarily agree to be part of this research study, to follow the study procedures, and to provide necessary information to the doctor, nurses, or other staff members, as requested.

Signature : Date :

(Patient)

| **Witness**  …………………………………………………………….  (Name)  …………………………………………………………….  (IC Number)  …………………………………………………………….  (Signature)  …………………………………………………………….  (Date) | **Researcher**  …………………………………………………………….  (Name)  …………………………………………………………….  (IC Number)  …………………………………………………………….  (Signature)  …………………………………………………………….  (Date) |
| --- | --- |

**BORANG KEIZINAN MENJALANI UJIAN FECAL OCCULT BLOOD**

**Tajuk Penyelidikan:**

Penilaian Kesan Minuman Susu Kultur ke atas Pesakit Parkinson yang Mengalami Sembelit.

**Tujuan Ujian Fecal Occult Blood**

Sebelum anda di bekalkan dengan susu kultur ini, Ujian fecal occult blood akan dilakukan kepada anda dan tujuan ujian ini di lakukan adalah untuk memastikan penyebab masalah sembelit anda adalah berpunca dari masalah saraf penyakit Parkinson dan bukan kerana penyakit lain seperti barah atau keradangan usus. Fecal occult blood test adalah ujian yang merangkumi pengambilan spesimen najis di dalam botol yang disediakan pihak makmal dan akan di hantar ke makmal HUKM bagi mengkaji kehadiran darah dalam najis. Jika ujian fecal occult blood ini positive, anda akan digugurkan dari menyertai penyelidikan ini dan seterusnya anda akan di rujuk ke pakar usus (gastroenterologist) bagi ujian endoscopic (colonoscopy) untuk rawatan dan tindakan selanjutnya.

**Nama Penyelidik:** Prof. Datin Dr. Norlinah Mohamed Ibrahim.

**Persetujuan**

Saya,….………………………………………………………., No Kad Pengenalan: ………………..…………….....

- telah membaca maklumat dalam Helaian Maklumat untuk Peserta **termasuk maklumat tentang risiko dalam penyelidikan ini .**
- telah diberi masa untuk memikirkan tentang penyelidikan ini dan semua pertanyaan daripada saya telah dijawab serta memenuhi kepuasan saya.
- faham bahawa saya bebas untuk menarik diri daripada penyelidikan ini pada bila-bila masa tanpa memberi sebab.
- faham bahawa identiti saya tidak akan didedahkan dalam laporan.

Saya, dengan ini bersetuju secara sukarela untuk mengambil bahagian dalam penyelidikan ini, mengikut prosedur kajian and memberi maklumat yang diperlukan kepada doktor, jururawat atau kakitangan.

Tandatangan : Tarikh:

| **Saksi**  …………………………………………………………….  (Nama)  …………………………………………………………….  (No. Kad Pengenalan)  …………………………………………………………….  (Tandatangan)  …………………………………………………………….  (Tarikh) | **Penyelidik**  …………………………………………………………….  (Nama)  …………………………………………………………….  (No. Kad Pengenalan)  …………………………………………………………….  (Tandatangan)  …………………………………………………………….  (Tarikh) |
| --- | --- |

**INFORMED CONSENT FORM FOR FECAL OCCULT BLOOD TEST**

**Research Title:** The effectiveness of Probiotics on Constipation in Parkinson Disease

**Purpose of Fecal Occult Blood Test**

Fecal occult blood refers to blood in the feces that is not visibly apparent. A fecal occult blood test checks for hidden blood in the stool. The test is done by collecting stool sample into a container provided by our lab.The purpose of this investigation is basically to rule out any other large bowel disease such as bowel polyps or malignancy. The test will be done to you before we provide the probiotics and if the fecal occult bood tested positive, you will be withdrawn from this study and you will be referred to gastroenterologist for endoscopic test (colonoscopy) and further assessment and management.

**Researcher’s Name:** Prof. Datin Dr. Norlinah Mohamed Ibrahim

**Consent:**

I, …………………………………………………………………………., IC No: ……………………..………………….....

- have read the information in the Patient Information Sheet **including information regarding the risk in this study**
- Have been given time to think about it and all of my questions have been answered to my satisfaction.
- Understand that I may freely choose to withdraw from this study at any time without given any reason.
- Understand that my anonymity will be ensured in the write-up.

I hereby, voluntarily agree to be part of this research study, to follow the study procedures, and to provide necessary information to the doctor, nurses, or other staff members, as requested.

Signature : Date :

(Patient)

| **Witness**  …………………………………………………………….  (Name)  …………………………………………………………….  (IC Number)  …………………………………………………………….  (Signature)  …………………………………………………………….  (Date) | **Researcher**  …………………………………………………………….  (Name)  …………………………………………………………….  (IC Number)  …………………………………………………………….  (Signature)  …………………………………………………………….  (Date) |
| --- | --- |
