# Supplementary material for: Multi-strain probiotics (Hexbio) containing MCP BCMC strains improved constipation and gut motility in Parkinson’s disease: A randomised controlled trial
Source: PLoS One. 2020 Dec 31;15(12):e0244680. doi: 10.1371/journal.pone.0244680 (PMC7774928; doi:10.1371/journal.pone.0244680)
Supplement: Attachment — Submitted filename: RESPONSE TO REVIEWERS.docx [file pone.0244680.s003.docx]

**13 November 2020**

**PONE-D-20-21349: Probiotic-prebiotic therapy improved constipation and gut motility in Parkinson’s disease: A Randomised controlled trial**

**Dear Academic Editor and Reviewers,**

**We have revised the manuscript as per comments and suggestions by the editor and reviewers. We present the point-to-point rebuttal in a table form.**

| **Experts** | **Comments** | **Authors action/ explanation** | **Changes in the manuscript** |
| --- | --- | --- | --- |
| **Staff Editor** | The key concerns noted by the reviewers relate to the limitations of the study sample and statistical analyses. In particular, Reviewer 3 noted the need for sensitivity analyses due to the high dropout rate, while Reviewer 1 highlighted a need for clarification about the sample size calculations. Furthermore, the reviewers raised concerns about multiple testing, via the multiple t-test analyses. These limitations have implications for the interpretation of the results. | Concerns regarding sample size, sensitivity analysis and multiple testing have been addressed based on each reviewer’s comment. Detailed actions and explanations are provided below according to each reviewer section. | Changed accordingly |
|  | Additionally, the reviewers requested more information about the name of the probiotic strains used in this study. | The names of the probiotics strain have been added in the manuscript (methods). Additionally, we have used the commercial name (Hexbio®) with MCP®BCMC®strains to replace probiotic-prebiotic therapy, to comply with the recent funder’s request on the description of the probiotics supplied. | Yes |
| **Journal requirements** | 1. Please ensure that your manuscript meets PLOS ONE's style requirements, including those for file naming. | We have amended the style according to PLOS ONE requirements. | Yes |
|  | 2. Thank you for submitting your clinical trial to PLOS ONE and for providing the name of the registry and the registration number. The information in the registry entry suggests that your trial was registered after patient recruitment began. PLOS ONE strongly encourages authors to register all trials before recruiting the first participant in a study.  As per the journal’s editorial policy, please include in the Methods section of your paper: 1) your reasons for your delay in registering this study (after enrolment of participants started); 2) confirmation that all related trials are registered by stating: “The authors confirm that all ongoing and related trials for this drug/intervention are registered”.  Please also ensure you report the date at which the ethics committee approved the study as well as the complete date range for patient recruitment and follow-up in the Methods section of your manuscript. | We have addressed these concerns in the methods section, stating reasons for delay.  We have also included a statement which says. “The authors confirm that all ongoing and related trials for this drug/intervention are registered”.  We have included the date when the ethics committee approved the study and complete date range for patient recruitment in the methods section. | Yes |
|  | 3. Thank you for stating the following in the Financial Disclosure section:  " NMI study received funding from Universiti Kebangsaan Malaysia Research Grant  The active treatment and placebo were provided free of charge by study sponsor (B-CROBES SDN BHD).  The funders had no role in study design, data collection and analysis, decision to publish or in the preparation of the manuscript"  We note that you received funding from a commercial source: B-CROBES.  Please provide an amended Competing Interests Statement that explicitly states this commercial funder, along with any other relevant declarations relating to employment, consultancy, patents, products in development, marketed products, etc.  Within this Competing Interests Statement, please confirm that this does not alter your adherence to all PLOS ONE policies on sharing data and materials by including the following statement: "This does not alter our adherence to PLOS ONE policies on sharing data and materials.” (as detailed online in our guide for authors http://journals.plos.org/plosone/s/competing-interests).  If there are restrictions on sharing of data and/or materials, please state these. Please note that we cannot proceed with consideration of your article until this information has been declared.    Please include your amended Competing Interests Statement within your cover letter. We will change the online submission form on your behalf.  Please know it is PLOS ONE policy for corresponding authors to declare, on behalf of all authors, all potential competing interests for the purposes of transparency. PLOS defines a competing interest as anything that interferes with, or could reasonably be perceived as interfering with, the full and objective presentation, peer review, editorial decision-making, or publication of research or non-research articles submitted to one of the journals. Competing interests can be financial or non-financial, professional, or personal. Competing interests can arise in relationship to an organization or another person. Please follow this link to our website for more details on competing interests: http://journals.plos.org/plosone/s/competing-interests | Thank you for this.  We have provided an amended Competing Interest Statement as required which complies all requirements by PLOS ONE, in the Cover Letter  **Amended Competing Interest statement**  The probiotics and the placebo were provided by B-CROBES SDN BHD. B-CROBES had no role in the study design, data collection and data analysis. On behalf of the authors, I declare that there are no competing interests, personal, professional, financial or non-financial related to the study or its publication. This does not alter our adherence to PLOS ONE policies on sharing data and materials. There are no restrictions on sharing of data and/or materials related to this study. | - |
|  | 4. We note that you have indicated that data from this study are available upon request. PLOS only allows data to be available upon request if there are legal or ethical restrictions on sharing data publicly. For information on unacceptable data access restrictions, please see <http://journals.plos.org/plosone/s/data-availability#loc-unacceptable-data-access-restrictions>.    In your revised cover letter, please address the following prompts:    a) If there are ethical or legal restrictions on sharing a de-identified data set, please explain them in detail (e.g., data contain potentially identifying or sensitive patient information) and who has imposed them (e.g., an ethics committee). Please also provide contact information for a data access committee, ethics committee, or other institutional body to which data requests may be sent.    b) If there are no restrictions, please upload the minimal anonymized data set necessary to replicate your study findings as either Supporting Information files or to a stable, public repository and provide us with the relevant URLs, DOIs, or accession numbers. Please see <http://www.bmj.com/content/340/bmj.c181.long> for guidelines on how to de-identify and prepare clinical data for publication. For a list of acceptable repositories, please see <http://journals.plos.org/plosone/s/data-availability#loc-recommended-repositories>.    We will update your Data Availability statement on your behalf to reflect the information you provide. | We have read PLOS ONE’s data sharing policy and we are happy to share de-identified data sets.  We have uploaded this in the listed repository Dryad with the following url. <https://doi.org/10.5061/dryad.np5hqbzr7> | Not applicable |
|  | 5. Please include captions for your Supporting Information files at the end of your manuscript, and update any in-text citations to match accordingly. Please see our Supporting Information guidelines for more information: http://journals.plos.org/plosone/s/supporting-information. | We do not have any supporting information. We have included all tables in the manuscript. |  |
| **Reviewer 1** | 1. The authors first state the sample size is computed for type I error 0.01 and 80% power and then 1 sentence later say it was computed with type I error rate 0.05 and 90% power. Which? | We apologize for this mistake. We have omitted the redundant and conflicting statement and have corrected the sample size based on 80% power with two-sided 5% significance, as was used in our study. Based on this, the new sample size required is 35. We have amended this is the manuscript as below:  *“The sample size was calculated using Power and Sample Size Calculation software [14], with two-sided 5% significance and 80% power based on a reference study by Sakai et al[15] which assessed constipation symptoms in a healthy population who consumed fermented milk containing probiotic. The authors reported 40% and 10% changes in the produced hard or lumpy stools (≥ 25% of bowel movements) after 3 weeks of treatment among the treatment and control group, respectively. Additional 10% per group was added to compensate for drop-outs and non-response, with a final sample of 35 patients per group. Nevertheless, a minimum of 25 patients per arm was selected for this study due to resource feasibility.* | Yes. |
|  | 2. The authors use reference 15 to justify sample size assumptions? What assumptions? What is a clinically relevant effect size here? What type of statistical test is this computation based on? | The computation was based on Power and Sample Size Calculation software (reference no 14). This program PS is applicable for alternative hypothesis of interest expressed as differing response rates. We calculated using two proportions of treatment and control groups. | We have added “The authors reported 40% and 10% changes in the produced hard or lumpy stools (≥ 25% of bowel movements) after 3 weeks of treatment among the treatment and control group, respectively”. |
|  | 3. a) While the authors talk about a binary constipation outcome, I am seeing 9 different ordinal outcomes,  b) with no adjustment for multiplicity. | a. We agree with the reviewer regarding the binary constipation outcomes assessment, while the outcomes were ordinal outcomes. We have corrected this in the manuscript.  Findings presentation and interpretation in Abstract and Results sections have been revised. The treatment effect and its corresponding 95% CI in Table 4 has been revised.  Various regression models in generalized linear models were used depending on the type of data for all outcomes (ie. ordinal, binary and linear regressions)  b. We agree with this comment and have made the necessary revision.  We applied the multiple correction for primary outcome with multiple outcomes only. Multiple correction was not conducted for secondary and exploratory outcomes. To address multiple correction: we have added a statement in the discussion part of the manuscript as follows:  *“However, changes between groups with good treatment effect for primary outcome which measured bowel opening frequency must be interpreted with caution, as it reached a significant corrected p value.”*  Reference: Li, G., Taljaard, M., Van Den Heuvel, E. R., Levine, M. A., Cook, D. J., Wells, G. A., Devereaux, P. J. & Thabane, L. 2016. An Introduction to Multiplicity Issues in Clinical Trials: The What, Why, When and How. International Journal of Epidemiology 46(2): 746-755. | ***Statistical analysis section*:** We have included the following statement “corrected *p* value of <0.005 was regarded as statistically significant for the primary outcome. The uncorrected *p* value <0.05 was reported for the gut motility and PD outcomes”  ***Findings in abstract and result section*:** Details of uncorrected and corrected p values have been added and reported accordingly in texts and Table 4 (treatment effect). The term “significantly” for outcomes especially the primary outcome which did not reach corrected *p* value has been omitted in the text.  ***Table 4*** has been revised accordingly. Foot notes has been added as follow:  “Primary outcome: significant at *p* <0.005; Gut motility and PD outcome outcomes: significant at *p* <0.05.”    ***Discussion*:** The following statement has been added to the final paragraph of the discussion section.  “*Additionally, despite a relatively small sample size, we were able to detect significant changes between groups with good treatment effect for both primary and secondary outcome parameters. However, these significant changes must be interpreted with caution, due to the small sample size.”* |
|  | 4. a) The authors indicate that they will need 30 in each group with a minimum of 25, yet they have only 22 in one group. Again, I question these numbers anyway, given the number of hypothesis tests done for the primary outcome. | We had initially recruited 55 PD patients with constipation into our study, of which 27 patients were in probiotic group while 28 patients in placebo group. As we were blinded to treatment assignment, we were unaware of the group that the dropouts belonged to.  Only after unblinding, we realized that the probiotics group had more dropouts(n=5) compared to the placebo group (n=2). We acknowledge that this imbalance in numbers of patients per group has certain limitations, which we have included in the discussion | Yes |
|  | 5. The conclusions should state whether the assumptions of your sample size computation were realized in the trial. There are numerous minor problems with verb tenses, plurals and singulars, and definite and indefinite articles that could be corrected easily by an editor. | We have included a statement in the conclusion regarding assumptions of sample size computation were not realised in the trial.  We have additionally corrected the grammatical issues with our manuscript. | Yes, in the conclusion as below:  *While, these findings strengthen existing evidence on the efficacy of probiotics on constipation in PD, the clinical benefits observed should be interpreted with caution due to limitations in achieving the required sample size* |
| **Reviewer 2** | 1. The term used "multi-strain probiotic and a prebiotic fiber" would mean symbiotic supplementation. I suggest that the authors use the term symbiotics, as it will define with greater accuracy the supplement offered in the essay. | Thank you for this helpful comment. We agree on the possible synbiotic mechanisms in the probiotic therapy used in this study. However, when we sought further clarification from the manufacturer, we were informed that the composition of prebiotic in this therapy was low (2%) and may not fulfil the criteria for synbiotic. As such, we have inferred to the possibility of a synbiotic mechanism but have not replaced the combination as synbiotic. In addition, we have used the commercial name, as requested by Reviewer 3 in the manuscript to refer to the probiotic used in the study. | We have amended the title to include the name of the commercial product and the strains used in the probiotic and have removed the word prebiotic.  Reference  Pandey KR, Naik SR, Vakil BV. Probiotics, prebiotics and synbiotics- a review. *J Food Sci Technol*. 2015;52(12):7577-7587. doi:10.1007/s13197-015-1921-1 |
|  | 2. The applied methodology is adequate and relevant, as well as the statistical analysis. However, when studying constipation, an analysis of food consumption and water consumption is essential, as this direct influence on the presence and severity of constipation is known. Another methodology that could be applied to estimate severity is the application of the bristol scale. | Thank you for this comment, which is very relevant. Unfortunately, we did not quantify the amount of water or food consumption in the study due to possibility of recall bias. We have included this in the limitation.  We agree that the Bristol Stool Scale to quantify could also be used to determine severity of constipation. We have addressed this in the limitation section. | Yes – Limitations |
|  | 3. Regarding the discussion, I believe it can be deepened based on the scientific bases described in the literature on the pathophysiological mechanisms of constipation. | Thank you. We have included a paragraph on this in the discussion. | Yes |
|  | 4. Regarding the limitations of the study, I believe it is important to explain the reasons for not being applied to assess water consumption and the Bristol scale. | We agree with this and have added these in the limitations. | Yes |
| **Reviewer 3** | 1- Type of substance- to allow replicability of the study, and to open a deeper discussion of the data, it is essential to authors to describe the species’ strain number of the bacteria included in the substance | We have now included the microbial composition of probiotics in the study drug composition section within the methodology. | Yes |
|  | 2- Is the substance used any type of commercial product? It is important to provide this information throughout the text. | Yes, the commercial product is known as Hexbio® containing MCP®BCMC® We have now added the commercial name as well throughout the manuscript. | Yes |
|  | 3- With respect to the statistical analysis choice- why the authors used multiple t-test analysis (or correspondent non-parametric tests), instead of choosing a simultaneous analysis (ANOVA and their corresponding non-parametric test)? Performing multiple t-test analysis, besides the lack of information about time-group interaction, it increases the risk of Type-I error. | We agree that there was multiple- t test (or corresponding non-parametric tests) used, instead of ANOVA and their corresponding non-parametric test) as comparisons were done between 2 groups only (Probiotic vs Placebo groups) in Table 2 and 3.  The multiple correction was not conducted, and uncorrected *p* value was applied for multiple- t test (or corresponding non-parametric tests) in Table 3. This was because the analysis involved secondary outcomes. |  |
|  | 4- a) Considering the short duration of the study, and the number of dropouts, the authors would perform a sensitivity analysis, carrying forward the initial data of the patients who dropout.  b) The existence of side-effects should not be neglected in the discussions. The use of FOS it is generally not well accepted by the elderly, due to the excessive gas formation, which can be an even greater complication in PD patients. | a) We agree with this. We have performed a sensitivity analysis by intention-to-treat in the Statistical analysis section. There were no changes in the findings for GTT difference which was conducted by carrying forward the initial data of the patients who dropout.  We have included this as a study limitation in the Discussion section.  b) We have included clearer information of the side effects. The abdominal bloating was transient and resolved with discontinuation of probiotics. | ***4a***  ***Statistical analysis section:*** we have revised the statement to “Analyses were done on an intention-to-treat basis, including all eligible patients who were randomised and who returned for a week-8 visit” for clarity.  ***Result section:*** Results has been revised according to initial recruitment data of 27 and 28 patients of Probiotic and Placebo groups, respectively. Changes were made in text, Table 2, Table 3 and Table 5.    ***Discussion:*** The following statements have been added for clarity.  “Finally, we could only perform the intention-to-treat analysis for one secondary outcome which was GTT difference. As for the rest of study outcomes, we were unable to perform intention-to-treat analysis as patients in both groups dropped out within the first week of trial and were not keen for further follow up. |
|  | 5- When discussing the limitation of the study due a lack of objective information on fiber intake, the authors could include the presence of other diet components also important to gut health, such as fatty acids, phytochemicals, and vitamins. All these components can serve as bacteria substrate and metabolites formation, which important to the gut functioning and metabolism. | Thank you for this helpful suggestion. We have added this in the limitations. | Yes |
|  | 6- How the authors found, from their study (in the abstract’s conclusion), the Type I evidence to the results? Type I evidence requires results from systematic reviews! | We have removed this statement from the abstract. | Yes |
| **Reviewer 4** | 1) The name of probiotic strains used in this study are missing throughout the manuscript - please amend accordingly | Thank you for this. We have included this in the methodology section of the manuscript | Yes |
|  | 2) Please discuss the potential bias in patients’ recruitment protocols based on Rome III criteria in light of current Rome IV criteria | We have explained regarding the potential bias on recruitment in light of Rome IV criteria in the limitation. | We have added this in the limitation as follows:  *“While the updated ROME IV may have facilitated an earlier diagnosis of constipation, we believe the ROME III used in our inclusion criteria was adequate to detect constipation in our patients, as constipation in PD is typically chronic and may even predate the motor symptoms. Hence we believe the use of ROME III would not have led to significant false negative or bias in the recruitment of patients[24].* |
|  | 3) Why Authors decided to choose Garrigues Questionnaire, as the low, positive likelihood ratios limit the clinical usefulness of this scale to identify individuals with constipation and there is no or scarce data on the reliability of the GQ or on its sensitivity to observe the change in bowel habits (McCrea et al. Review article: self-report measures to evaluate constipation. Aliment Pharmacol Ther 2008; 27(8): 638-48) | We agree with this statement. We have explained this in detail in the discussion. The GQ was chosen as it was designed in 1999, as a self-report questionnaire for constipation based on Rome III criteria.  However, we realize that it is not the best tool to detect changes in constipation severity over time, which may have contributed to the negative findings on self-reported symptoms. We have explained this in the discussion. | We have included a paragraph in the discussion to address this |
|  | 4) The results are promising but still the number of individuals included in this RCT is low, which limits its generalisability | Yes, we realize that this is an important limitation and explained that the generalisability of this study may be limited by the sample size, although our findings were similar to that by Baricella et al. which had a larger sample size.  We have added a statement in the conclusion which states this:  *“Multi-centre studies with larger sample size are required to validate our findings and if the positive effects could be generalised to other populations with differing dietary background.”* |  |
